# Grape Seed Procyanidin Extract (GSPE) Improves Goat Sperm Quality When Preserved at 4 °C

**DOI:** 10.3390/ani9100810

**Published:** 2019-10-15

**Authors:** Fei Wen, Yu Li, Tianyu Feng, Yeqing Du, Fa Ren, Likun Zhang, Ning Han, Shulan Ma, Fangzhou Li, Peng Wang, Jianhong Hu

**Affiliations:** 1 Key Laboratory of Animal Genetics, Breeding and Reproduction of Shaanxi Province, College of Animal Science and Technology, Northwest A & F University, Yangling 712100, Shaanxi, China; 13087561604@163.com (F.W.); yuli0912@126.com (Y.L.); fengtianyu0105@126.com (T.F.); duyeqing1026@126.com (Y.D.); renfa0306@126.com (F.R.); zhanglikun6@163.com (L.Z.); hn126320@126.com (N.H.); 18702993635@163.com (S.M.); juvenile02@163.com (F.L.); 2 Ningxia Key Laboratory of Cerebrocranial Diseases, School of Basic Medical Science, Ningxia Medical University, Yinchuan 750004, China

**Keywords:** grape seed procyanidin extract (GSPE), liquid preservation, goat semen, sperm quality

## Abstract

**Simple Summary:**

Artificial insemination (AI) is widely used in goats, stimulating the development of semen preservation techniques. Oxidative stress is considered to be the main cause of sperm quality decline over time. In this study, we explored the effect of grape seed procyanidin extract (GSPE) during the liquid preservation of goat semen. The results showed that adding GSPE into the basic diluent enhanced sperm quality by eliminating oxidative stress. The most suitable concentration for the preservation of goat semen at 4 °C was 30 mg/L. This work suggests that promotes the viability of goat semen stored at low temperatures and provides a theoretical foundation for the development of more efficient diluents.

**Abstract:**

Grape seed procyanidin extract (GSPE) has been shown to possess antioxidative effects. This experiment was designed to study the effect of GSPE during the liquid storage of goat semen. Semen samples were collected from six sexually mature goats. The samples were treated with different concentrations of GSPE (10, 30, 50, and 70 mg/L) in basic diluent and stored at 4 °C for 120 h; samples without GSPE were used as the control group. The results showed that sperm motility, acrosome membrane integrity, mitochondrial activity, plasma membrane integrity, total antioxidative capacity (T-AOC), catalase (CAT) activity, and superoxide dismutase (SOD) activity in the treatment groups were significantly higher than in the control group, whereas malondialdehyde (MDA) content was lower than in the control group (*p* < 0.05). In the treatment group, sperm quality in the 30 mg/L GSPE group was significantly higher than the other groups (*p* < 0.05). Furthermore, artificial insemination (AI) results showed that litter sizes were higher in the 30 mg/L GSPE group than in the control group (*p* < 0.05). In summary, this experiment showed that adding GSPE to the basic diluent improved sperm quality and that 30 mg/L of GSPE was the most suitable concentration for the liquid preservation of goat semen at 4 °C.

## 1. Introduction

The use of artificial insemination (AI) in animal breeding is now widespread. AI is a more economical, simpler, and a more successful technique than embryo transfer or natural mating for the rapid dissemination of elite livestock genetics [1]. Semen preservation which is a the key step in AI, prolongs storage time and maintains fertilization capacity, facilitates long-distance transportation, and improves the reproductive efficiency of elite male animals [2,3]. One of two sperm preservation protocols, liquid or frozen, may be employed for AI [4]. Liquid preservation results in better conception rates than frozen [5,6]; however, as preservation time increases, the conception rate decreases. This decline is due to the gradual deterioration of sperm quality, including sperm motility, mitochondrial activity, acrosome and plasma membrane integrity [4]. Oxidative stress is thought to be the main cause of the decline in sperm quality decline owing to the activities of reactive oxygen species (ROS) during the storage period [1]. Mammalian sperm contain large amounts of unsaturated fatty acids, which are susceptible to ROS attack [7,8,9]. ROS causes lipid peroxidation, DNA damage, increased membrane permeability, and even reduces reproductive capacity [5,10]. There are many reports regarding the antioxidant properties used for semen preservation, such as the use of *Lycium barbarum*, *Laminaria japonica* polysaccharides [1], and glycine [6]. In vivo, the seminal plasma contains sufficient antioxidants to maintain ROS balance [11,12]. However, the balance becomes disrupted during semen preservation in vitro, which results in the production and accumulation of large amounts of ROS [10]. Therefore, the application of antioxidants to prolong semen quality during preservation is important. 

Grape seed procyanidin extract (GSPE) are derived from grape seeds, a group of polyphenolic bioflavonoids [13,14]. GSPE has a wide range of pharmacological and therapeutic effects. Hamsters fed with GSPE, have reduced levels of oxidative stress [15]. Furthermore, GSPE prevents ethanol-induced DNA damage in mouse brain cells through its antioxidation activity [16], and protects against H_2_O_2_-induced DNA damage in Fao cells [13]. Furthermore, GSPE reduces oxidative stress and enhances sperm motility in nickel sulfate-induced rat teste [17]. However, there are no reports to date on the utility of GSPE in semen preservation. Therefore, in this study, GSPE was introduced into the diluent to investigate its effect on the quality of goat sperm stored at 4 °C. Sperm motility, acrosome integrity, mitochondrial activity, plasma membrane integrity, total antioxidative capacity (T-AOC), superoxide dismutase (SOD) activity, catalase (CAT) activity, and malondialdehyde (MDA) levels were studied. In addition, the pregnancy rate of goats was used as an important indicator. Our research aimed to provide a scientific basis for the development of GSPE as a new supplement in the cryopreservation of goat semen. 

## 2. Material and Methods

### 2.1. Reagents 

All chemicals in this study were purchased from Sigma Chemical Co. (St. Louis, MO, USA). 

### 2.2. Goat Semen Collection 

A total of 60 ejaculates (10 ejaculates per goat) were collected twice a week from six sexually mature goats using an artificial vagina. Samples were subjectively evaluated. Only ejaculates with a sperm concentration ≥3.0 × 10^9^ spermatozoa/mL, motility ≥ 80%, and normal sperm morphology ≥90% were included [1]. To eliminate the variability among individuals, all ejaculates were pooled. All procedures in the study strictly followed the guideline and regulation of the Chinese Experimental Animal Society. 

### 2.3. Goat Semen Preservation 

All pooled ejaculates were divided into five equal fractions. The control fraction was diluted with basic preservation media (1.15 g/L glucose, 1.45 g/L D-fructose, 0.25 g/L PVA, 0.23 g/L ethylenediaminetetraacetic acid (EDTA), 1.17 g/L sodium citrate, and 0.125 g/L sodium hydrogen carbonate). The remaining fractions were diluted in basic preservation media containing different concentrations of GSPE (10, 30, 50, or 70 mg/L). All fractions were diluted to 6.0 × 10^8^ spermatozoa/ml. After dilution, the semen was cooled from 37 °C to 4 °C at a rate of 0.2 to 0.3 °C/min and stored in a refrigerator for 120 h at 4 °C. 

### 2.4. Sperm Motility

Semen samples (5 μL) were selected from the 0–120 h-preserved specimens. All samples were incubated at 37 °C for 5 min. Samples were diluted with phosphate buffered saline (PBS) containing bovine serum albumin (1 mg/mL) to a count of 20 × 10^6^ spermatozoa/ml at 37 °C before analysis [18]. The semen (5 μL) was then placed on a preheated slide, and covered with a coverslip. Sperm motility was detected by CASA (HVIEW-SSAV version 12.1; Hamilton Thorne, Beverly MA, USA) at 60 HZ (an image sampling frequency). Five fields of view were randomly selected for the analysis of sperm motility on each slide. There were at least three replicates in each group. 

### 2.5. Sperm Functional Plasma Membrane Integrity

Sperm functional plasma membrane integrity was assessed by hypoosmotic swelling test (HOST) as described by Lodhi et al. [19]. The preserved semen (50 μL) was mixed with 1 mL (150mOsmol kg ^−1^) HOST (7.35g/L sodium citrate dihydrate and 13.51 g/L fructose) and placed in a water bath at 37 °C for 30 min. After incubation, semen (15 μL) was dripped onto a glass slide. Sperm tails were observed under a light microscope (400×). In each experiment, at least 200 sperm were observed and recorded to calculate the number with swollen and curled tails. Sperm with curled tail curled or that were swollen were recorded o have intact membranes.

### 2.6. Acrosomal Membrane Integrity 

Sperm acrosome integrity was assessed by the fluorescein isothiocyanate-conjugated peanut agglutinin (FITC-PNA) staining as described by Ren et al. [1]. The semen (30 μL) was fixed in anhydrous methanol for 10 min, and fully covered with the FITC-PNA (30 μL) at 37 °C for 30 min in the dark. After incubation, the semen was washed three times with phosphate buffered saline (PBS) for 10 min and then mounted with anti-fading solution. Acrosomal integrity was evaluated under a fluorescence microscope with a DMX Nikon digital camera (Tokyo, Japan). Under a fluorescence microscope, sperm acrosomes show bright green fluorescence, and such sperm were recorded as having an intact acrosome. There were at least three replicates in each group.

### 2.7. Mitochondrial Activity

Sperm mitochondrial activity was evaluated by dual fluorescent staining, rhodamine 123 (Rh123), and propidium iodide (PI) staining, as described by Ren et al. [1]. Briefly, Rh123 solution (3µL) was mixed with 1 mL semen and incubated at 37 °C for 20 min in the dark. Then semen was stained with PI (10 μL, 0.5 mg/mL PBS) and incubated at 37 °C for 10 min. Next, semen was centrifuged at 600× *g* for six min, the supernatant was discarded. The sperm pellets were resuspended in 1 ml PBS. Finally, an aliquot of each sample (10 μL) was dropped onto a glass slide and observed under a fluorescence microscope (400×). Each group had five replicates and no less than 200 sperm were counted per slide. Sperm cells with functional mitochondrial activity stained with Rh123 and showed a reddish brown color under the fluorescence microscope.

### 2.8. Measurement of T-AOC, SOD Activities, CAT Activities, and MDA Level

Two-hundred microliters of liquid preserved semen was centrifuged at 1600× *g* for 5 min. After discarding the supernatant, 600 μL of Triton X-100 (1%) was added to the sperm pellet for enzyme extraction for 20 min. Subsequently, the resuspended semen were centrifuged at 4000× *g* for 30 min at 25 °C, and the supernatant was collected as the crude extract of the enzymes for follow-up determination. The T-AOC, SOD activity, CAT activity, and MDA levels were measured using assay kits (Nanjing Jiancheng Bioengineering Institute, Jiangsu, China) according to the manufacturer’s instructions. The plate was read by a spectrophotometer (Shanghai Spectrophotometer Co. Ltd., China) at 520 nm (T-AOC), 560 nm (SOD activity), 520 nm (CAT activity), and 532 nm (MDA level). There were at least three technical duplications in all groups. 

### 2.9. Fertility Trials 

Semen samples (stored for 72 h) in the control group, and in the 30 mg/L GSPE group, preservation was used for artificial insemination. Glass tubes were used to store the diluted semen. A single infusion of 0.25 mL contained 2.5 × 10^8^ spermatozoa. A total of 163 goats were used in the experiment, 83 of which served as controls. The estrous cycle was synchronized using intravaginal sponges impregnated with 45 mg of fluorogestone acetate (FGA) for 11 days. On the ninth day, goats were treated with a single intramuscular injection of equine chorionic gonadotropin (eCG, 300 IU) and cloprostenol (125 g). Each goat was inseminated twice. Initial intracervical insemination was performed at 11–12 h after estrus, and a second insemination was performed 10 h later. Pregnancy was determined by ultrasonography at 30 days after AI. Following birth, litter size was recorded and analyzed. 

### 2.10. Statistical Analysis

The results were expressed as mean ± standard deviation, and statistical differences were analyzed by Statistical Product and Service Solutions 22.2 software (SPSS Inc., Chicago, IL, USA). The differences in mean values were assessed using Duncan’s multiple range tests and one-way analysis of variance (ANOVA). *p* < 0.05 was considered significant. Pregnancy rate and litter sizes were analyzed by the chi-squared test and the *t*-test. 

## 3. Results 

### 3.1. Sperm Motility

The effect of varying GSPE concentrations on the motility of the liquid stored goat sperm at 4 °C is depicted in Table 1. The results showed that the sperm motility did not differ significantly between the GSPE preservation and control groups at 0 h, 24 h, and 48 h. However, with ongoing preservation to 120 h, sperm motility rapidly decreased in the control. Sperm motility in those samples preserved with GSPE at 30 mg/L was highest at 72 h, 96 h, and 120 h (*p* < 0.05) (Table 1). 

### 3.2. Functional Plasma Membrane Integrity 

The effects of different doses of GSPE on sperm functional plasma membrane integrity upon preservation goat semen at 4 °C are depicted in Table 2. The results showed that there was no significant difference between the GSPE and control groups at 0 h. At 24 h, GSPE appeared to prevent sperm functional plasma membrane damage; this was significant at the GSPE 30 mg/L level compared with the control (*p* < 0.05). This phenomenon was similar from 48 h to 120 h.

### 3.3. Acrosome Integrity 

The effects of varying GSPE concentrations on goat sperm acrosome integrity during liquid storage at 4 °C are shown in Table 3. No significant difference in sperm acrosome integrity was found between the GSPE and control groups from 48 h to the 120 h. GSPE maintained sperm acrosome integrity, in particular, GSPE at 30 mg/l significantly rescued semen acrosome integrity compared with the control (*p* < 0.05). This pattern was similar at 48 h to 120 h.

### 3.4. Mitochondrial Activity 

The effects of varying GSPE concentrations on goat sperm mitochondrial activity during liquid storage at 4 °C are shown in Table 4. There was no significant difference in sperm mitochondrial activity between the GSPE preservation and control groups at 0 h. In each timeline, GSPE is maintained, being highest in the 30 mg/L GSPE group (*p* < 0.05). 

### 3.5. Analysis of Antioxidant Activities

**Figure 1:**
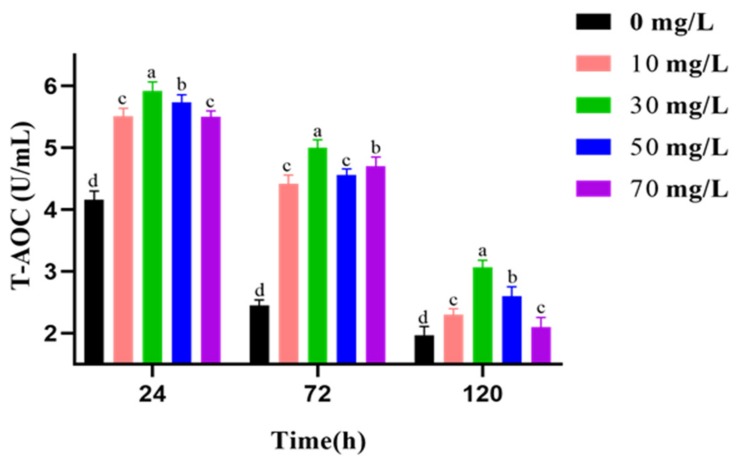
Effect of grape seed procyanidin extracts (GSPE) on the total antioxidative capacity (T-AOC) of goat sperm of preserved at 4 °C. Within the same incubation time, values with different superscripts differ among treatments (*p* < 0.05). The values are expressed as mean ± SEM.

The effects of varying GSPE concentrations on T-AOC of goat sperm activity during liquid storage at 4 °C are shown in Figure 1. The results show that T-AOC was spontaneously decreased with preservation time. GSPE greatly boosted T-AOC from 24 h to 120 h. In particular, T-AOC was best preserved in the 30 mg/L GSPE group. 

**Figure 2:**
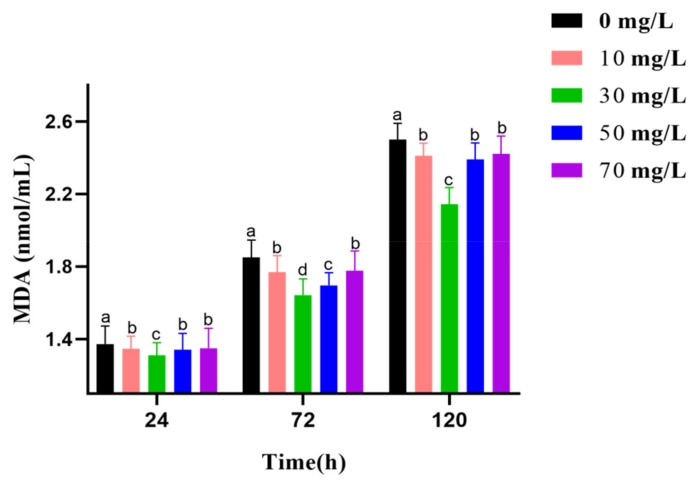
Effect of grape seed procyanidin extracts (GSPE) on the MDA level of goat sperm of preserved at 4 °C. Within the same incubation time, values with different superscripts differ among treatments (*p* < 0.05). The values are expressed as mean ± SEM.

The results for the effect of GSPE on MDA level are shown in Figure 2. With prolonged preservation, the MDA level increased. At 24 h, GSPE slightly prevented MDA production. However, at 72 h and 120 h, the 30 mg/L GSPE impeded MDA production. In particular, MDA levels in 30 mg/L GSPE group is the lowest compared to other groups at 24 h, 72 h, and 120 h. 

**Figure 3:**
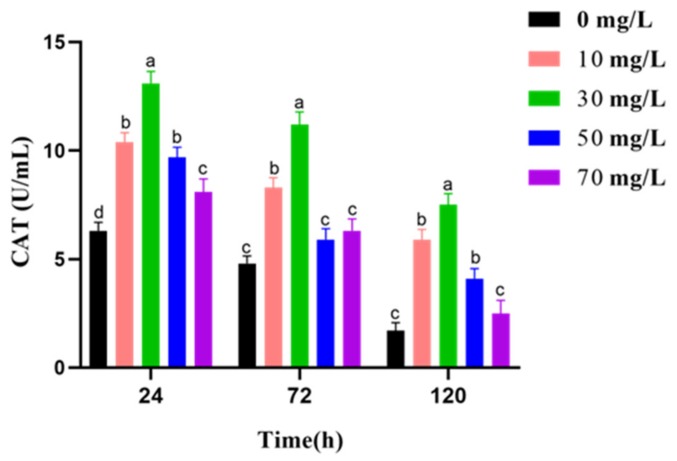
Effect of grape seed procyanidin extracts (GSPE) on the CAT activity of goat sperm of preserved at 4 °C. Within the same incubation time, values with different superscripts differ among treatments (*p* < 0.05). The values are expressed as mean ± SEM.

In Figure 3, CAT activity shows a slight decline with prolonged the preservation. However, the treatment groups show more higher CAT activity than control groups in respective preservation time. Most importantly, the activity of CAT is highest in the 30 mg/L GSPE group at 24 h, 72 h, and 120 h.

**Figure 4:**
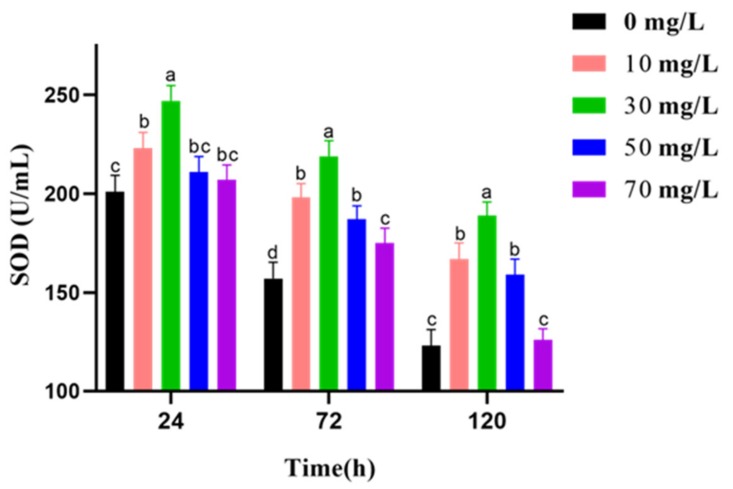
Effect of grape seed procyanidin extracts (GSPE) on the SOD activity of goat sperm of preserved at 4 °C. Within the same incubation time, values with different superscripts differ among treatments (*p* < 0.05). The values are expressed as mean ± SEM.

The effects of varying GSPE concentrations on the SOD activity of goat semen activity during liquid storage at 4 °C are shown in Figure 4. The results showed that the SOD activity decreased with prolonged preservation. GSPE therefore enhanced the increase in SOD activity in respective preservation phases. Nevertheless, SOD activity in the GSPE (30 mg/L) groups is highest between 24 h and 120 h. 

### 3.6. Pregnancy Rates and Litter Sizes

The results showed no significant difference in pregnancy rate between the control group and the GSPE (30 mg/L) group, rates were 68.7% and 72.5%, respectively (Table 5). However, the litter size in the GSPE group (1.47 ± 0.21 ^b^) was significantly higher than in the control group (1.50 ± 0.30 ^a^, *p* < 0.05 Table 5). 

## 4. Discussion 

Considerable progress in semen preservation has been made over the past few decades; however, the decline that occurs in sperm quality during this process has not been resolved [5,11,20]. During in vitro preservation, there is a risk of oxidative damage to sperm owing to ROS accumulation [8,12]. The mammalian sperm membrane contains a large amount of polyunsaturated fatty acids (PUFA). When ROS is excessive, the PUFA of the sperm membrane is attacked, which causes lipid peroxidation (LPO) [10,20]. LPO of sperm membranes can also lead to failure of the acrosome reaction [20,21]. Excessive ROS damages cellular proteins, and the lipid structure of the cell membrane, furthermore excessive ROS causes gene mutations and accelerates cell death [10]. Previous studies have shown that the addition of antioxidants can significantly improve sperm quality in the rat [22], bull [11], dog [12], boar [8], and ram [5] by reducing ROS accumulation during the preservation process. GSPE has been reported to have a strong antioxidant capacity [13,15,16,23]. The results of this study demonstrated that the addition of GSPE to the extender enhanced goat sperm quality and antioxidant capacity during liquid preservation at 4 °C. To the best of our knowledge, this study is the first to report that GSPE can be preserved in a rotating liquid at a 4 °C by enhancing antioxidant capacity. 

Sperm motility, sperm plasma membrane integrity, and acrosome integrity are important for the acrosome reaction and further penetration through the zona pellucida of the ovum [1,24,25]. Mitochondria play a key role in maintaining normal sperm function and energy homeostasis through oxidative phosphorylation and ATP synthase [26]. In this experiment, the quality of sperm was decreased as the semen preservation time increased, and the accumulation of ROS lead to a loss in membrane selectivity, permeability, and membrane change, as well as a reduction in activity and vigor. However, the results showed that the addition of GSPE significantly improved sperm motility, sperm plasma membrane integrity, acrosome integrity and mitochondrial activity compared to control. Similarly, Zhao et al. [27] note that GSPE treatment significantly enhances rat sperm motility and morphology by attenuating cisplatin (DDP)-induced oxidative stress. Su et al. [17] indicate that GSPE improves rat sperm motility by offsetting Ni-induced apoptosis and oxidative stress. Semen quality does decline with increased storage time, and oxidative damage is considered an important factor affecting semen quality [10]. Structurally, GSPE has a hydroxyl group conjugated with double bonds, which is the basis for GSPE as an oxygen radical scavenger and lipid peroxidation inhibitor [14,28]. Previous studies have shown that goat sperm motility is 24.7% after three days of storage at 5 °C [29]. In this study, sperm motility reached 68.59% after three days of liquid preservation, and GSPE significantly improve sperm motility in liquid preservation. Mammalian spermatozoa membrane is very sensitive to ROS attack as they are rich in polyunsaturated fatty acids (PUFA). The process of preservation results in over production of reactive oxygen species, which is extremely detrimental to spermatozoa. ROS promote peroxidation of lipids, resulting in intracellular oxidative burden. The sequence of events involves lipid peroxidation, loss of membrane integrity with increased permeability, reduced sperm motility, structural DNA damage, and apoptosis [7,11]. Many previous studies have confirmed that this increase in ROS levels reduces sperm quality owing to imbalances in the antioxidant defense system [5,8,11,12]. SOD and CAT are important components of the antioxidant system in seminal plasma, which can antagonize and block free radicals [30,31]. ROS not only causes cell damage through the peroxidation of PUFA in biofilms, but also causes cell damage through the decomposition products of LPO. MDA levels indirectly reflect the extent to which cells are attacked by free radicals [32]. 

To further explore the effect of GSPE on the preservation of goat semen at 4 °C, T-AOC, CAT activity, SOD activity, and MDA level were evaluated. The results showed that T-AOC, CAT activity, and SOD activity in the GSPE groups are higher than in the control groups (*p* < 0.05), whereas MDA levels in the GSPE groups were significantly lower than in the control groups (*p* < 0.05). Chen et al. [31] report that treatment with GSPE enhances SOD and GSH-Px activity and reduces MDA content in the oxidative damage of murine kidneys. A previous study reports that GSPE reverses the decreased activity of antioxidant enzymes (SOD and GSH-Px) and elevates levels of oxidative stress in rat testes [23]. Su et al. [17] suggest that GSPE counteracts oxidative stress in rat testis by directly decreasing MDA and NO, via the scavenging of H₂O₂. In addition, GSPE rescues DNA from oxidative damage by removing the free radicals, such as ROS and –OH [33]. GSPE can effectively scavenge superoxide anion radicals and hydroxyl radicals, thus interrupting free radical chain reaction [34]. BiS et al. [35] suggest that GSPE can play an antioxidant role by enhancing the expression of antioxidant enzymes and reducing the regeneration of free radicals. Our findings are consistent with the studies described above, showing that GSPE can eliminate oxidative damage by enhancing the activity of antioxidant enzymes. 

In this study, the addition of GSPE enhanced sperm quality and the optimal concentration required to achieve this effect was 30 mg/L. It is worth noting that when the concentration exceeded 30 mg/l, the physiological properties and antioxidant enzyme activities of sperm decreased. We suggest that high concentrations of GSPE may cause sperm apoptosis, but the specific molecular mechanisms requires further research. Finally, we used AI to further verify the effect of GSPE supplementation. Analysis of the pregnancy rates in the experiment indicated that the 30 mg/L GSPE group (72.5%) was not significantly different from in the control group (68.7%). The litter size in the GSPE group (1.50 ± 0.30 ^a^) was significantly greater than in the control group (1.47 ± 0.21 ^b^) (*p* < 0.05). Fang et al. [7] found that the addition of Iodine methionine (IM) to the dilution significantly increase litter size, but the pregnancy rate is not significantly different compared to control. This is similar to our research results. The lack of difference in pregnancy rate could be a consequence of sperm dose and its association with compensable sperm abnormalities. Previous studies have shown that sperm quality is positively correlated with litter size [36]. Su et al. [17] show that GSPE attenuates oxidative stress damage in mouse testis through the Nrf2 signaling pathway and thus improves reproductive capacity. These observations indicated that GSPE increases litter size by improving sperm quality. The results of the current experiments combined with previous studies suggest that GSPE can improve fertility and strong antioxidants are thought to play a major role. 

## 5. Conclusions

The present study revealed that GSPE improved the quality of goat sperm, in particular, GSPE at 30 mg/L elicited optimal effects upon the preservation of goat semen at 4 °C. We consider that the antioxidant capacity of GSPE is an important factor in the improvement of sperm quality.

## Figures and Tables

**Table 1:** Effect of grape seed procyanidin extracts (GSPE) on the motility of goat sperm of preserved at 4 °C (%).

Time (h)	GSPE (mg/L)				
	0	10	30	50	70
0	85.29 ± 1.22	84.79 ± 1.42	85.00 ± 1.31	84.65 ± 1.23	84.50 ± 1.32
24	78.31 ± 1.31	78.60 ± 1.19	78.98 ± 1.40	78.29 ± 1.33	78.94 ± 1.60
48	70.12 ± 1.44	70.13 ± 1.53	70.73 ± 0.69	70.51 ± 1.55	70.45 ± 1.12
72	66.80 ± 1.62 ^c^	68.59 ± 1.12 ^b^	69.45 ± 0.77 ^a^	68.31 ± 1.04 ^b^	66.21 ± 1.34 ^c^
96	54.30 ± 1.29 ^e^	58.32 ± 1.23 ^c^	65.34 ± 1.04 ^a^	61.58 ± 1.17 ^b^	56.44 ± 1.69 ^d^
120	45.32 ± 1.11 ^d^	46.89 ± 1.64 ^c^	51.46 ± 0.97 ^a^	49.41 ± 1.20 ^b^	45.77 ± 1.31 ^d^

Within the same incubation time, values with different superscripts differ among treatments (*p* < 0.05). The values are expressed as mean ± SEM.

**Table 2:** Effect of grape seed procyanidin extracts (GSPE) on the plasma membrane integrity of goat sperm of preserved at 4 °C (%).

Time (h)	GSPE (mg/L)				
	0	10	30	50	70
0	74.32 ± 1.27	74.32 ± 0.79	75.54 ± 1.31	74.67 ± 1.25	74.51 ± 1.77
24	70.69 ± 1.63 ^c^	71.84 ± 1.55 ^c^	73.82 ± 1.28 ^a^	72.71 ± 1.98 ^b^	71.43 ± 1.11 ^c^
48	64.25 ± 1.42 ^e^	77.72 ± 1.03 ^a^	70.43 ± 0.77 ^b^	68.08 ± 1.23 ^c^	67.01 ± 1.03 ^d^
72	60.20 ± 1.51 ^d^	65.11 ± 1.01 ^b^	67.13 ± 1.51 ^a^	65.85 ± 1.01 ^ab^	63.02 ± 1.20 ^c^
96	53.05 ± 0.88 ^e^	56.31 ± 1.22 ^b^	58.74 ± 1.01 ^a^	56.88 ± 1.44 ^b^	55.71 ± 1.25 ^b^
120	44.32 ± 1.16	47.43 ± 1.13 ^c^	50.97 ± 0.75 ^a^	48.42 ± 1.02 ^b^	46.33 ± 1.34 ^c^

Within the same incubation time, values with different superscripts differ among treatments (*p* < 0.05). The values are expressed as mean ± SEM.

**Table 3:** Effect of grape seed procyanidin extracts (GSPE) on the acrosome integrity of goat sperm of preserved at 4 °C (%).

Time (h)	GSPE (mg/L)				
	0	10	30	50	70
0	85.26 ± 1.35	84.83 ± 1.34	85.16 ± 1.42	85.34 ± 1.24	84.76 ± 1.43
24	81.85 ± 1.61 ^b^	81.73 ± 1.22 ^b^	83.19 ± 1.33 ^a^	82.54 ± 1.63 ^ab^	80.55 ± 1.21 ^c^
48	78.94 ± 1.48 ^c^	79.45 ± 1.12 ^bc^	81.49 ± 1.41 ^a^	80.79 ± 1.52 ^ab^	79.43 ± 1.22 ^bc^
72	72.50 ± 1.62 ^d^	75.13 ± 1.32 ^c^	78.46 ± 1.04 ^a^	76.11 ± 1.02 ^b^	74.40 ± 1.04 ^c^
96	65.69 ± 1.31 ^e^	69.45 ± 0.88 ^c^	74.00 ± 1.11 ^a^	70.33 ± 1.17 ^b^	67.72 ± 1.31 ^d^
120	62.05 ± 1.22 ^c^	65.02 ± 1.26 ^b^	67.55 ± 0.65 ^a^	65.33 ± 0.51 ^b^	64.75 ± 1.06 ^b^

Within the same incubation time, values with different superscripts differ among treatments (*p* < 0.05). The values are expressed as mean ± SEM.

**Table 4:** Effect of grape seed procyanidin extracts (GSPE) on the mitochondrial activity of goat sperm of preserved at 4 °C (%).

Time (h)	GSPE (mg/L)				
	0	10	30	50	70
0	87.60 ± 1.23	87.27 ± 1.14	87.24 ± 1.51	87.34 ± 1.23	87.53 ± 1.86
24	77.11 ± 1.31	78.80 ± 1.13 ^bc^	81.12 ± 1.23 ^a^	80.12 ± 1.21 ^ab^	78.34 ± 1.64 ^bc^
72	62.79 ± 1.45 ^c^	64.99 ± 1.01 ^b^	70.36 ± 1.02 ^a^	65.20 ± 1.32 ^b^	63.79 ± 1.11 ^c^
120	43.19 ± 1.44 ^d^	47.37 ± 1.04 ^bc^	50.78 ± 1.13 ^a^	48.03 ± 0.62 ^b^	46.72 ± 1.20 ^c^

Within the same incubation time, values with different superscripts differ among treatments (*p* < 0.05). The values are expressed as mean ± SEM.

**Table 5:** Comparisons of pregnancy rates 30 days after artificial insemination (AI) using preserved goat semen (72 h at 4 °C) and litter sizes between control group and 30 mg/L GSPE group.

Groups	Hybridization Number	Conception Number	Conception Rate (%)	Litter Sizes
Control	83	57	68.7%	1.47 ± 0.21 ^b^
30 Mg/L GSPE Group	80	58	72.5%	1.50 ± 0.30 ^a^

^a,b^ Values within a column with different superscripts are significantly different (*p* < 0.05). The values are expressed as mean ± SEM.

## References

[1] Ren F., Fang Q., Feng T.Y., Li Y., Wang Y.H., Zhu H.J., Hu J.H. (2019). Lycium barbarum and Laminaria japonica polysaccharides improve Cashmere goat sperm quality and fertility rate after cryopreservation. Theriogenology.

[2] Casali R., Pinczak A., Cuadro F., Guillen-Munoz J.M., Mezzalira A., Menchaca A. (2017). Semen deposition by cervical, transcervical and intrauterine route for fixed-time artificial insemination (FTAI) in the ewe. Theriogenology.

[3] Zhao B.T., Han D., Xu C.L., Luo M.J., Chang Z.L., Tan J.H. (2009). Protocol optimization for long-term liquid storage of goat semen in a chemically defined extender. Reprod. Domest. Anim..

[4] O’Hara L., Hanrahan J.P., Richardson L., Donovan A., Fair S., Evans A.C.O., Lonergan P. (2010). Effect of storage duration, storage temperature, and diluent on the viability and fertility of fresh ram sperm. Theriogenology.

[5] Paul R.K., Balaganur K., Bahire S.V., Kumar D., Singh R. (2018). Supplementation of cauda epididymal plasma improves sperm characteristics following liquid preservation of ram semen at 3–5 °C. Reprod. Fertil. Dev..

[6] Maxwell W.M., Salamon S. (1993). Liquid storage of ram semen: A review. Reprod. Fertil. Dev..

[7] Fang Q., Wang J., Hao Y.Y., Li H., Hu J.X., Yang G.S. (2017). Effects of iodine methionine on boar sperm quality during liquid storage at 17 °C. Reprod. Domest. Anim..

[8] Li H., Zhang X.G., Fang Q., Liu Q., Du R.R., Yang G.S. (2017). Supplemental effect of different levels of taurine in modena on boar semen quality during liquid preservation at 17 °C. Anim. Sci. J..

[9] Barrios B., Pérez-Pé R., Gallego M., Tato A., Osada J., Muino-Blanco T., Cebrian-Perez J.A. (2000). Seminal plasma proteins revert the cold-shock damage on ram sperm membrane. Biol. Reprod..

[10] Aitken R.J. (2017). Reactive oxygen species as mediators of sperm capacitation and pathological damage. Mol. Reprod. Dev..

[11] Bilodeau J.F., Blanchette S., Gagnon C., Sirard M.A. (2001). Thiols prevent h2o2-mediated loss of sperm motility in cryopreserved bull semen. Theriogenology.

[12] Domosławska A., Zdunczyk S., Franczyk M., Kankofer M., Janowski T. (2018). Selenium and vitamin e supplementation enhances the antioxidant status of spermatozoa and improves semen quality in male dogs with lowered fertility. Andrologia.

[13] Llópiz N., Puiggròs F., Céspedes E., Arola L., Ardévol A., Bladé C., Salvado M.J. (2004). Antigenotoxic effect of grape seed procyanidin extract in fao cells submitted to oxidative stress. J. Agric. Food Chem..

[14] Bagchi D., Garg A., Krohn R.L., Bagchi M., Tran M.X., Stohs S.J. (1997). Oxygen free radical scavenging abilities of vitamins c and e, and a grape seed proanthocyanidin extract in vitro. Res. Commun. Mol. Pathol. Pharmacol..

[15] Décordé K., Teissèdre P.L., Sutra T., Ventura E., Cristol J.P., Rouanet J.M. (2009). Chardonnay grape seed procyanidin extract supplementation prevents high-fat diet-induced obesity in hamsters by improving adipokine imbalance and oxidative stress markers. Mol. Nutr. Food Res..

[16] Guo L., Wang L.H., Sun B., Yang J.Y., Zhao Y.Q., Dong Y.X. (2007). Direct in vivo evidence of protective effects of grape seed procyanidin fractions and other antioxidants against ethanol-induced oxidative dna damage in mouse brain cells. J. Agric. Food Chem..

[17] Su L., Deng Y., Zhang Y., Li C., Zhang R., Sun Y. (2011). Protective effects of grape seed procyanidin extract against nickel sulfate-induced apoptosis and oxidative stress in rat testes. Toxicol. Mech. Methods.

[18] Mata-Campuzano M., Soleilhavoup C., Tsikis G., Martinez-Pastor F., de Graaf S.P., Druart X. (2015). Motility of liquid stored ram spermatozoa is altered by dilution rate independent of seminal plasma concentration. Anim. Reprod. Sci..

[19] Lodhi L.A., Zubair M., Qureshi Z.I., Ahmad I., Jamil H. (2008). Correlation between hypo-osmotic swelling test and various conventional semen evaluation parameters in fresh Nili-Ravi buffalo and Sahiwal cow bull semen. Pak. Vet. J..

[20] Olivera-Muzante J., Fierro S., Gil J. (2011). Conception rates in ewes after ai with ram semen preserved in milk-egg yolk extenders supplemented with glycerol. Reprod. Domest. Anim..

[21] Rodrigues M.A.M., Souza C.E.A., Martins J.A.M., Rego J.P.A., Oliveira J.T.A., Domont G. (2013). Seminal plasma proteins and their relationship with sperm motility in santa ines rams. Small Rumin. Res..

[22] Güvenç M., Cellat M., Gökçek İ., Yavaş İ., Yurdagül Özsoy Ş. (2019). Effects of thymol and carvacrol on sperm quality and oxidant/antioxidant balance in rats. Arch. Physiol. Biochem..

[23] Wang Y., Chen F., Liang M., Chen S., Zhu Y., Zou Z. (2018). Grape seed proanthocyanidin extract attenuates varicocele-induced testicular oxidative injury in rats by activating the nrf2-antioxidant system. Mol. Med. Rep..

[24] Fernandes G.H., De Carvalho P.T., Serra A.J., Crespilho A.M., Peron J.P., Rossato C. (2015). The effect of low-level laser irradiation on sperm motility, and integrity of the plasma membrane and acrosome in cryopreserved bovine sperm. PLoS ONE.

[25] Sutovsky P., Navara C.S., Schatten G. (1996). Fate of the sperm mitochondria, and the incorporation, conversion, and disassembly of the sperm tail structures during bovine fertilization. Biol. Reprod..

[26] Ruiz-Pesini E., Díez-Sánchez C., López-Pérez M.J., Enriquez J.A. (2007). The role of the mitochondrion in sperm function: Is there a place for oxidative phosphorylation or is this a purely glycolytic process?. Curr. Top. Dev. Biol..

[27] Zhao Y.M., Gao L.P., Zhang H.L., Guo J.X., Guo P.P. (2014). Grape seed proanthocyanidin extract prevents ddp-induced testicular toxicity in rats. Food Funct..

[28] Foo L.Y. (1981). Proanthocyanidins: Gross chemical structures by infrared spectra. Phytochemistry.

[29] Qiu J.H., Li Y.W., Xie H.L., Li Q., Dong H.B., Sun M.J. (2016). Effects of glucose metabolism pathways on sperm motility and oxidative status during long-term liquid storage of goat semen. Theriogenology.

[30] Gadea J., Sellés E., Marco M.A., Coy P., Matás C., Romar R., Ruiz S. (2004). Decrease in glutathione content in boar sperm after cryopreservation: Effect of the addition of reduced glutathione to the freezing and thawing extenders. Theriogenology.

[31] Chen Q., Zhang R., Li W.M., Niu Y.J., Guo H.C., Liu X.H., Zhao L.J. (2013). The protective effect of grape seed procyanidin extract against cadmium-induced renal oxidative damage in mice. Environ. Toxicol. Pharmacol..

[32] Fraczek M., Szkutnik D., Sanocka D., Kurpisz M. (2001). peroxidation components of sperm lipid membranes in male infertility. Ginekol. Pol..

[33] Wood J.E., Senthilmohan S.T., Peskin A.V. (2002). Antioxidant activity of procyanidin-containing plant extracts at different phs. Food Chem..

[34] Fracassetti D., Costa C., Moulay L., Tomás-Barberán F.A. (2013). Ellagic acid derivatives, ellagitannins, proanthocyanidins and other phenolics, vitamin c and antioxidant capacity of two powder products from camu-camu fruit (myrciaria dubia). Food Chem..

[35] Bagchi D., Bagchi M., Stohs S.J., Das D.K., Ray S.D., Kuszynski C.A., Joshi S.S., Pruess H.G. (2000). Free radicals and grape seed proanthocyanidin extract: Importance in human health and disease prevention. Toxicology.

[36] Okazaki T., Mihara T., Fujita Y., Yoshida S., Teshima H., Shimada M. (2010). Polymyxin b neutralizes bacteria-released endotoxin and improves the quality of boar sperm during liquid storage and cryopreservation. Theriogenology.

